# Structural and functional characterization of endothelial microparticles released by cigarette smoke

**DOI:** 10.1038/srep31596

**Published:** 2016-08-17

**Authors:** Karina A. Serban, Samin Rezania, Daniela N. Petrusca, Christophe Poirier, Danting Cao, Matthew J. Justice, Milan Patel, Irina Tsvetkova, Krzysztof Kamocki, Andrew Mikosz, Kelly S. Schweitzer, Sean Jacobson, Angelo Cardoso, Nadia Carlesso, Walter C. Hubbard, Katerina Kechris, Bogdan Dragnea, Evgeny V. Berdyshev, Jeanette McClintock, Irina Petrache

**Affiliations:** 1 The Departments of Medicine, Indiana University School of Medicine, Indianapolis, IN, USA; 2 National Jewish Health, Denver, CO, USA; 3 Department of Biochemistry & Molecular Biology, Indiana University School of Medicine, Indianapolis, IN, USA; 4 Departments of Biochemistry & Molecular Biology and Pediatrics, Indiana University School of Medicine, Indianapolis, IN, USA; 5 Department of Chemistry, Indiana University, Bloomington, IN, USA; 6 Department of Pharmacology, Johns Hopkins University, Baltimore, MD, USA; 7 Department of Medicine, University of Illinois, Chicago, IL, USA; 8 Richard L. Roudebush Veteran Affairs Medical Center, Indianapolis, USA.

## Abstract

Circulating endothelial microparticles (EMPs) are emerging as biomarkers of chronic obstructive pulmonary disease (COPD) in individuals exposed to cigarette smoke (CS), but their mechanism of release and function remain unknown. We assessed biochemical and functional characteristics of EMPs and circulating microparticles (cMPs) released by CS. CS exposure was sufficient to increase microparticle levels in plasma of humans and mice, and in supernatants of primary human lung microvascular endothelial cells. CS-released EMPs contained predominantly exosomes that were significantly enriched in let-7d, miR-191; miR-126; and miR125a, microRNAs that reciprocally decreased intracellular in CS-exposed endothelium. CS-released EMPs and cMPs were ceramide-rich and required the ceramide-synthesis enzyme acid sphingomyelinase (aSMase) for their release, an enzyme which was found to exhibit significantly higher activity in plasma of COPD patients or of CS-exposed mice. The *ex vivo* or *in vivo* engulfment of EMPs or cMPs by peripheral blood monocytes-derived macrophages was associated with significant inhibition of efferocytosis. Our results indicate that CS, via aSMase, releases circulating EMPs with distinct microRNA cargo and that EMPs affect the clearance of apoptotic cells by specialized macrophages. These targetable effects may be important in the pathogenesis of diseases linked to endothelial injury and inflammation in smokers.

The release of heterogeneous (0.1–5 μm) microparticles (MPs) from plasma membranes into the extracellular space following cell activation or apoptosis contributes to inter-cellular communication, with implications in inflammation, thrombosis, and angiogenesis^1^,^2^,^3^. Endothelial microparticles (EMPs) are increased in the blood of individuals exposed to cigarette smoke (CS) who develop emphysema, a form of COPD characterized by permanent enlargement of airspaces^4^,^5^,^6^. Since not all smokers develop emphysema, these findings implicate EMPs as potential biomarker of disease risk, phenotype, or severity. However, a precise structural and functional characterization of EMPs released in response to CS is lacking and the mechanism of EMPs release is not known.

Circulating MPs (cMPs) comprise endothelium- as well as leukocyte- or platelet-derived MPs. EMPs released in response to CS may include exosomes (30–150 nm), membrane particles (100 nm–1 μm), and/or apoptotic vesicles (1–5 μm)^7^. Along with size, several other markers distinguish these populations. For example, exosomes, unlike apoptotic vesicles, originate intracellular and while devoid of histones^1^, they express CD63 and transport RNA/ microRNAs^8^,^9^,^10^. Whereas externalization of phosphatidylserine (PS) has been identified on both apoptotic bodies and exosomes, it is absent on membrane particles. A precise characterization of MPs types released following exposure to soluble components of CS that reach circulation following CS inhalation has not been reported.

Since excessive apoptosis of pulmonary capillary endothelial cells in response to CS is a central mechanism of alveolar wall destruction^11^,^12^, we interrogated the role of signaling pathways typically involved in endothelial apoptosis in EMPs release, such as the activation of acid sphingomyelinase (aSMase). This interest was further spurred by a recent report demonstrating that aSMase was necessary and sufficient for MPs release from glial cells^13^. aSMase is responsible for the production of ceramide in cholesterol-rich membrane microdomains by catalyzing the hydrolysis of sphingomyelin. Typically activated by various stressors, including oxidative stress and CS^14^,^15^, ceramide production is involved in both apoptosis^11^,^16^ and inflammatory activation^12^ of lung endothelial cells. Furthermore, recent reports implicate ceramide production in plasma membrane repair such as extrusion and elimination of patches of damaged plasma membranes, in an attempt to mitigate injury^17^,^18^. This led us to interrogate the role of aSMase in the mechanism of CS-induced EMPs release.

The biological function of CS-induced EMPs is unknown. The release of ceramide-enriched EMPs may account for increased ceramides in the plasma of smokers^19^ or in other conditions characterized by endothelial apoptosis. In turn, miRNA-carrying exosomes could account for paracrine or epigenetic signaling associated with pulmonary and systemic vascular damage or other biological activities relevant to COPD pathogenesis^20^. Similar to apoptotic cells, the burden of cMPs plasma levels is determined by the balance between MPs release and MPs clearance by the monocyte-macrophage system^21^,^22^. MPs themselves may impact monocyte-macrophage function, but this effect appears to be specific to the cell type from which MPs originate. For example, MPs released from apoptotic platelets promote an anti-inflammatory macrophage phenotype^22^, whereas MPs released from monocytes enhance peripheral blood monocytes derived macrophage (PBMDM)’s engulfment of antibody-coated red blood cells^23^, a pro-inflammatory trait. Since the role of CS-induced EMPs has not been reported yet, we focused our investigation on their impact on specialized phagocytes. Others and we have shown that CS and ceramides decrease apoptotic cell clearance by macrophages (efferocytosis), *via* alterations of the cytoskeleton and impaired intercellular membranes fusion^24^. We therefore hypothesized that MPs released during CS exposure will inhibit PBMDM efferocytosis.

Using human primary human lung endothelial cells, mouse models, and plasma samples from human subjects, we demonstrate that endothelial cells release ceramide-rich apoptotic and exosomal EMPs in response to CS *via* a mechanism that involves the activation of aSMase. Furthermore, CS-induced EMPs and mouse cMPs carry specific miRNA cargo signatures and are functionally active, impairing PBMDMs and splenic macrophages efferocytosis function.

## Results

### Microparticles released by CS are enriched in exosomes and membrane particles

**Figure 1:**
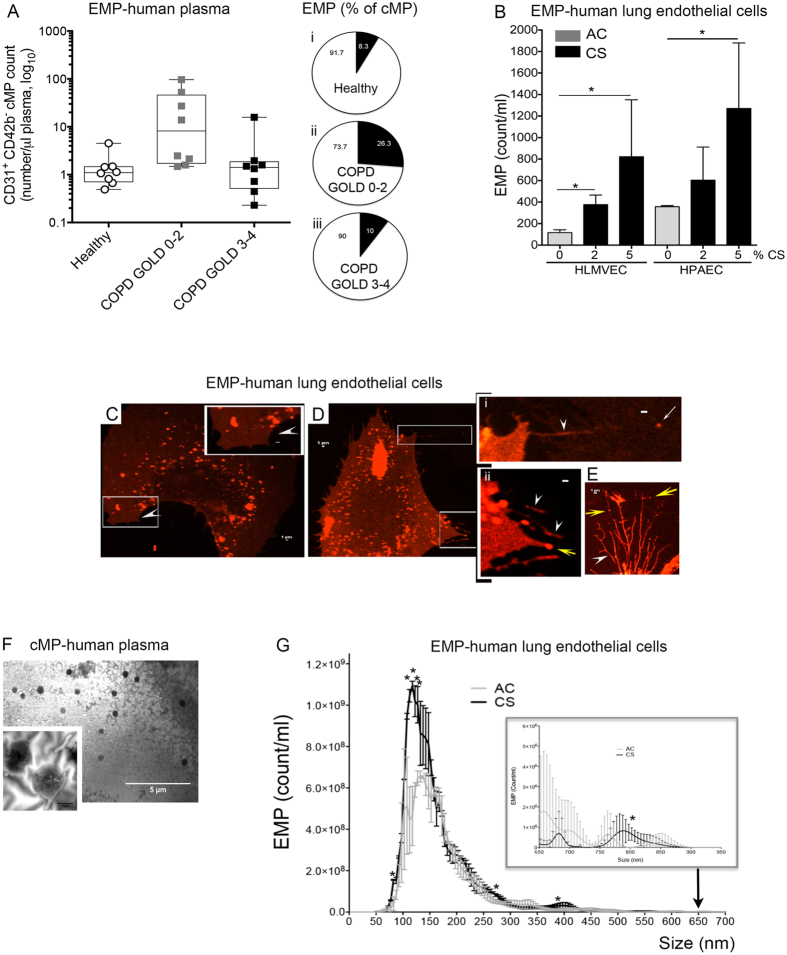
Characterization of microparticles released by CS exposure. (**A**) Abundance of circulating endothelial microparticles (EMPs) in human plasma (demographics in Supplementary Table 1). Data represent log-transformed CD31+/CD42b- events. ANOVA (p < 0.05; Bartlett’s *p < 0.0001). (**B**) Abundance of EMPs released from primary human lung microvascular (HLMVEC) and human pulmonary artery (HPAEC) endothelial cells exposed to CS or ambient air (AC) extract (v:v %; 2 h). Mean + SEM; ANOVA (p < 0.001; Tukey’s *p < 0.05, n = 5). (**C**–**E**) Representative images (n = 3) from videos (in Supplementary material) of HLMVEC transduced with plasma membrane-RFP-BacMan and exposed to AC ((**C)** protruding lamellipodia: arrowhead; insert); or to CS (5%, 1 h, (**D)** and insets; or 2%, 30 min, (**E)**). Note EMPs (yellow arrow) released from the plasma membrane at tips of retracting filopodia (arrowhead). (**F)**. Transmission electron microscopy micrograph of heterogeneous human plasma cMPs. (**G**). Abundance and size distribution of EMPs released from AC- and CS-exposed HLMVEC (5%; 2 h) measured by NanoSight-NS300 (Mean+/−SD; n =  3). CS released mostly EMPs 50 nm–200 nm (mode 129 nm; exosome fraction). t-test *p < 0.05 for sizes 82–92 nm, 107–127 nm, 262–277 nm, 372–387 nm, and 790–820 nm.

We first determined that our methodology of MPs isolation was consistent with previous reports of increased EMP in plasma of COPD patients^4^,^5^,^6^. Under an IRB-approved protocol, we isolated MPs using freshly collected plasma from healthy non-smoker controls (n = 8) and smokers with or without COPD, stratified by severity using GOLD 2007 (n = 17, details in Supplementary Table 1). Following platelet depletion and an ultraspeed centrifugation step, we quantified EMPs using flow cytometry, defining EMPs as CD31^+^/CD42b^−^ events. In this small cohort, we identified significant increases in circulating EMPs in smokers and those with mild COPD, when compared to healthy non-smokers (Fig. 1A). Consistent with previous reports using the largest and best characterized patient cohort^6^, those with advanced COPD had fewer circulating EMPs. In addition to the increased absolute values, we also measured marked relative increases of EMPs (as % of total cMPs) in smokers and mild COPD compared to healthy individuals (Fig. 1A inset).

We next investigated in cell cultures and mouse models whether direct exposure to soluble CS components expected to be absorbed in the circulation is sufficient to release EMPs from lung endothelium, independent of complex circulatory mediators that may be related to other structural alveolar cell (e.g. epithelial) injury, inflammation, or other factors associated with a chronic condition such as COPD. We exposed primary human lung microvascular endothelial cells (HLMVEC) obtained from non-smokers to soluble components of CS at previously determined non-lethal concentrations of aqueous CS extract^25^. Compared to cells exposed to ambient air extract as control (AC), CS exposure (2–5%, 2 h) doubled the EMPs release from both HLMVEC and from primary human pulmonary artery endothelial cells (HPAEC) (Fig. 1B). We then used time-lapse microscopy to directly visualize the EMPs release during CS exposure (1 h) of HLMVEC. Following transfection with red fluorescence protein targeting the myristoylation/palmitoylation sequence of lymphocyte-specific protein tyrosine kinase, we could visualize in red the plasma membrane of endothelial cells (Fig. 1C–E and Supplementary videos 1–3). In contrast to control cells that occasionally extended lamellipodia following addition of AC, those exposed to CS contracted their body, releasing EMPs mostly from tips of retracting filopodia-like structures. Similarly, cMPs isolated from the plasma of volunteers visualized by transmission electron microscopy (TEM; Fig. 1F) demonstrated membrane-bordered structures in the submicron size range. While these methods allowed visualization of MPs released by CS, they may not be sufficiently sensitive to detect exosomes. To more accurately define the size of CS-induced EMPs, we used NanoSight NS300 technology, which quantified the vast majority of CS-induced EMPs from HLMVEC as having diameters of 50 nm to 200 nm with a mode of 129 nm, which overlaps with exosomes size range of 30–150 nm (Fig. 1G). We also detected a doubling in larger size microparticles released in response to CS, e.g. 262–277 nm, 372–387 nm, and 790–820 nm (Fig. 1G, and inset), classifying them as membrane particles^1^, consistent with our microscopy data. Taken together, these results suggest that CS exposure releases a mixed population of both exosomes and membrane particles.

To ensure that findings obtained in primary humans cells were not cell-culture specific we corroborated these data with a controlled *in vivo* CS exposure model in mice and *ex-vivo* mouse lung endothelial cell (MLEC) studies, that allowed for further mechanistic interrogations. The cMPs abundance in plasma of C57BL6/J mice markedly and significantly increased following CS exposure, compared to littermate mice exposed to ambient air (Supplementary Figure 1A). Since mouse cMPs encompass circulating EMPs and MPs released by other cells (e.g. inflammatory cells), we investigated if, similarly to human cells, primary mouse lung endothelial cells (MLEC) release more EMPs in response to CS exposure. There was a significant increase in EMPs released from 10%CS-exposed MLEC which reached ~3-fold after 24 h (Supplementary Figure 1B). The population of EMPs released from MLEC likely contained a mixed population as it did in human cells, based on several complementary methods. First, sizing Nile-Red labeled EMPs against fluorescent polystyrene microbeads using flow cytometry (Supplementary Figure 1B Inset) identified membrane particles. Flow cytometry indicated that CS-induced EMPs had markedly increased histone expression (Supplementary Figure 1C), phosphatidylserine (PS) externalization (detected by annexin V staining, Supplementary Figure 1D), and CD63 (Supplementary Figure 1E). Whereas during control conditions, only half of the CD63-expressing EMPs were positive for PS (Supplementary Figure 1E inset), following CS exposure, almost all CD63-expressing EMPs co-expressed PS (Supplementary Figure 1E inset). These results suggest that CS exposure of MLEC, similar to that of HLMVEC releases a mixed population of apoptotic membrane particles and exosomes.

### Microparticles released by CS are enriched in miRs let-7d, −126, −125-5p, −22, and −191

**Figure 2:**
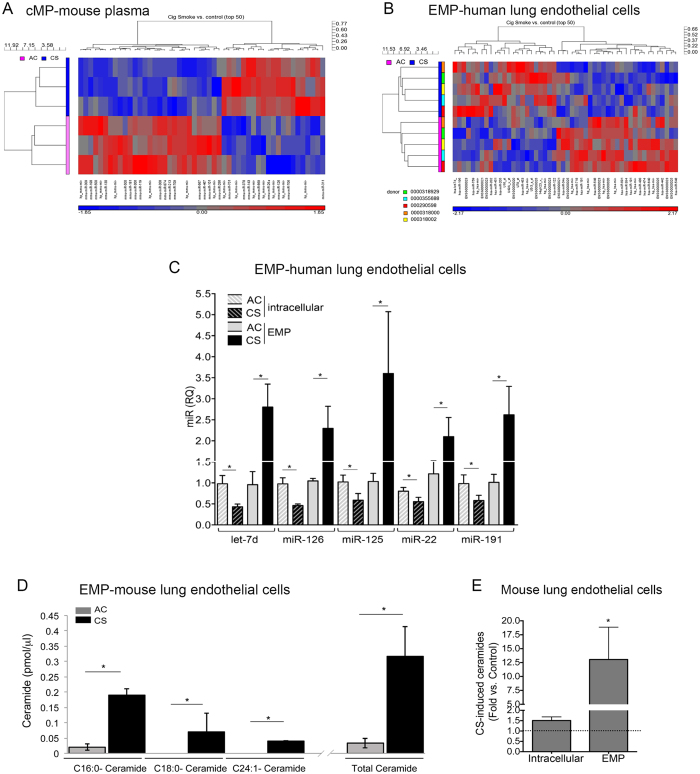
CS exposure increases specific miRNAs and ceramide species in mouse cMPs and human EMPs. (**A,B)** miRNA expression profiles in MPs. Heatmaps of top 50 individual differentially expressed miRNAs (Increased=red; decreased = blue) detected in cMPs from plasma of AC (pink, n = 3) or CS (3 d; blue, n = 3)-exposed mice (**F**); or in EMPs from AC (pink; n = 5) or CS (2%, 2 h; blue, n = 5)-exposed HLMVEC isolated from five donors. (**C)** miRNAs levels measured by RT-PCR intracellular and in EMPs from AC- and CS-exposed HLMVEC (Mean+SEM, n = 4, ANOVA p < 0.05; Sidak’s *p < 0.05)**. (D)** The most abundant ceramide species and total ceramide levels in EMP released from MLEC cells treated with CS (10%; 24 h) or AC control. Levels measured by mass spectrometry, normalized by volume of supernatant used to isolate EMP). Mean ± SEM; Student’s t-test *p < 0.05, n =  3. (**E)** Relative change in ceramide (vs. AC; dotted line) in intracellular- compared to EMPs compartments in CS (10%; 24 h)-exposed MLEC. Mean+SEM (t-test *p < 0.05, n =  3).

To further confirm the intracellular origin of the majority of CS-induced EMPs, we investigated if they carry microRNA (miRNA) and whether CS distinctly packages certain miRNA for export in EMPs. EMPs were isolated from supernatants of CS-exposed HLMVEC (n = 5 non-smoking donors, 5%, 2 h) and miRNA was detected in EMPs following total RNA isolation and hybridization to miRNA arrays. CS exposure of HLMVEC significantly increased the abundance of several miRNAs released in EMPs (Supplementary Table 2; heat map in Fig. 2A), the most affected being let-7d, which increased by more than 2-fold in EMPs released by CS, compared to control EMPs. Using a similar approach, we identified that the most upregulated circulating miRNAs in the mouse cMPs fraction following CS (heat map in Fig. 2B) were miR-142, miR-126, and miR-706 (Supplementary Table 3). We next interrogated which exosomal miRNAs that originate from the pulmonary endothelium are significantly enriched in the systemic circulation. Since miRNAs are highly conserved, and lung-specific EMPs cannot be isolated from a pool of cMPs, we performed meta-analysis of microarray data sets obtained from human cell EMPs and mouse cMPs. The metaanalysis identified the miRNAs let-7d, −126, −125-5p, and −22, as the most significantly up-regulated by CS exposure in both lung cell-derived EMPs and mouse cMPs (Table 1), suggesting their potential pulmonary endothelial origin in the systemic circulation. Several miRNAs, such as miR-3960 were not regulated by CS, but were highly abundant in both EMPs and cMPs (Supplementary Table 3). Validation of CS-induced increases in miR let-7d, 126, 125-5p, and 22 in EMPs detected by microarrays was accomplished with real time qPCR, to which we added measurement of miR-191, the circulating levels of which were reported increased by CS in human plasma^26^. Since control (housekeeping) miRNAs have not been validated yet for exosomes, we used the same starting RNA material (mass), in which we spiked-in *Caenorhabditis elegans* miRNA cel-mir-39, as endogenous control. For the detection of all miRNAs, a C_T_ < 35 was set as cut-off value. All the above miRNAs were selectively enriched in EMPs, but not intracellular, where their levels were actually significantly decreased (Fig. 2C). This suggested that CS-induced miRNAs dispatching in EMPs is selective, rather than nonspecifically reflecting intracellular miRNA profiles.

### Microparticles released by CS are enriched in C16- and C24-ceramides

We have previously shown that CS increases ceramides in mouse lungs^27^ and in cultured HLMVEC and MLEC^11^,^16^, where they act as mediators or effectors of cell stress responses, including apoptosis. Moreover, we unexpectedly detected lipophilic ceramide species in aqueous cell cultures supernatants and acellular BAL fluid^28^. We hypothesized that ceramides released from cells may be carried in aqueous environments by MPs. Interestingly, CS-released EMPs from MLEC were >10-fold enriched in ceramides compared to control EMPs (Fig. 2D). The relative increase in ceramide in EMPs (10.8-fold) following CS was significantly larger than their intracellular increase (1.6 fold), measured within the same timeframe (Fig. 2E), suggesting enrichment of ceramide in EMPs. The ceramide species identified in EMPs were similar to those within cells, with C16-ceramide and C24-ceramide predominaing (Supplementary Table 4). The distribution of ceramide species in cMPs isolated from human plasma was similar to that in whole (unfractionated) human plasma (Supplementary Figure 2A–C), suggesting that cMPs may be important carriers of ceramides in plasma of smokers and COPD individuals.

### Acid sphingomyelinase (aSMase) is required for CS-induced microparticle release

**Figure 3:**
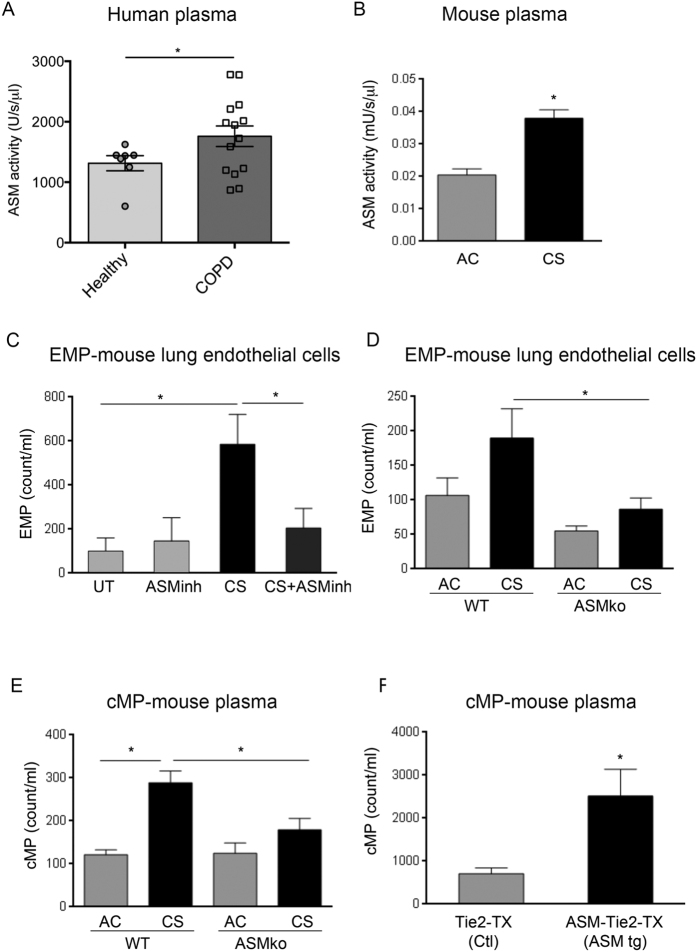
Role of acid sphingomyelinase (aSMase) in the release of CS-induced MPs. (**A)** ASM activity in plasma of healthy controls (n = 6) or individuals with COPD (n = 14; Supplementary Table 1). Mean+SEM (t-test *p < 0.05). (**B)** ASM activity in plasma of mice exposed to CS (3 h) or ambient air. Mean ± SEM; (t-test, *p < 0.01, n =  6). (**C**) EMPs released by MLEC exposed to CS (10%; 24 h) treated with a pharmacological inhibitor of aSMase (ASMinh; imipramine, 50 μM; 1 h). Mean+SEM (ANOVA p < 0.05; Tukey’s, *p < 0.05, n =  3). (D) EMPs released by MLEC isolated from aSMase deficient mice (ASMko; *Smpd1*^−/−^) exposed *ex vivo* to CS (10%; 24 h); Mean+SEM; ANOVA p = 0.02 (Sidak’s, *p < 0.05, n = 6). (E) Circulating MP (cMPs) released in plasma during CS exposure (3 h) of WT or ASMko mice. Mean + SEM; ANOVA p = 0.01 (Sidak’s *p < 0.05, n = 4–5). (**G**) cMPs released in plasma following induction of endothelial cell-specific aSMase expression in mice, using tamoxifen (TX, 3 d). Comparison is made between control mice (single transgenics expressing only Tie2::CRE) and aSMase-overexpressing mice (double transgenic, expressing Tie2::CRE-ASMase < flox>; ASM tg). Mean ± SEM (t-test, *p < 0.05, n = 3).

The increase in EMP ceramides and the rapid kinetics of cMPs release following CS exposure *in vivo* led us to investigate the aSMase pathway of ceramide-synthesis, an enzymatic pathway that is rapidly activated by stress. Coinciding with the timing of cMPs release, we measured increased soluble aSMase activity in plasma of human subjects (Fig. 3A) and mice exposed to CS (Fig. 3B). Since endothelial cells exhibit the most abundant levels of aSMase and can contribute to soluble aSMase levels in plasma, we interrogated whether endothelial aSMase is involved in CS-induced EMP release. Treatment with an aSMase inhibitor (imipramine) markedly decreased CS-induced EMPs from MLEC (Fig. 3C). We next isolated primary MLEC from *Smpd1*^−/−^ mice or wild type littermate and propagated them in culture. Following *ex vivo* CS exposure (10%, 24 h), aSMase-null endothelial cells produced significantly fewer EMPs compared to wild type endothelial cells (Fig. 3D). *In vivo*, transgenic mice with complete loss of aSMase (*Smpd1*^−/−^) released significantly fewer cMPs in plasma following CS exposure (3 h) compared to wild type mice (*Smpd1*^+/+^) (Fig. 3E). In contrast, cMP abundance was increased in plasma of mice following endothelial-specific conditional transgenic overexpression of aSMase (Fig. 3F). These findings suggest that aSMase activation is necessary for CS-induced EMP release.

### Microparticles inhibit macrophages efferocytosis

**Figure 4:**
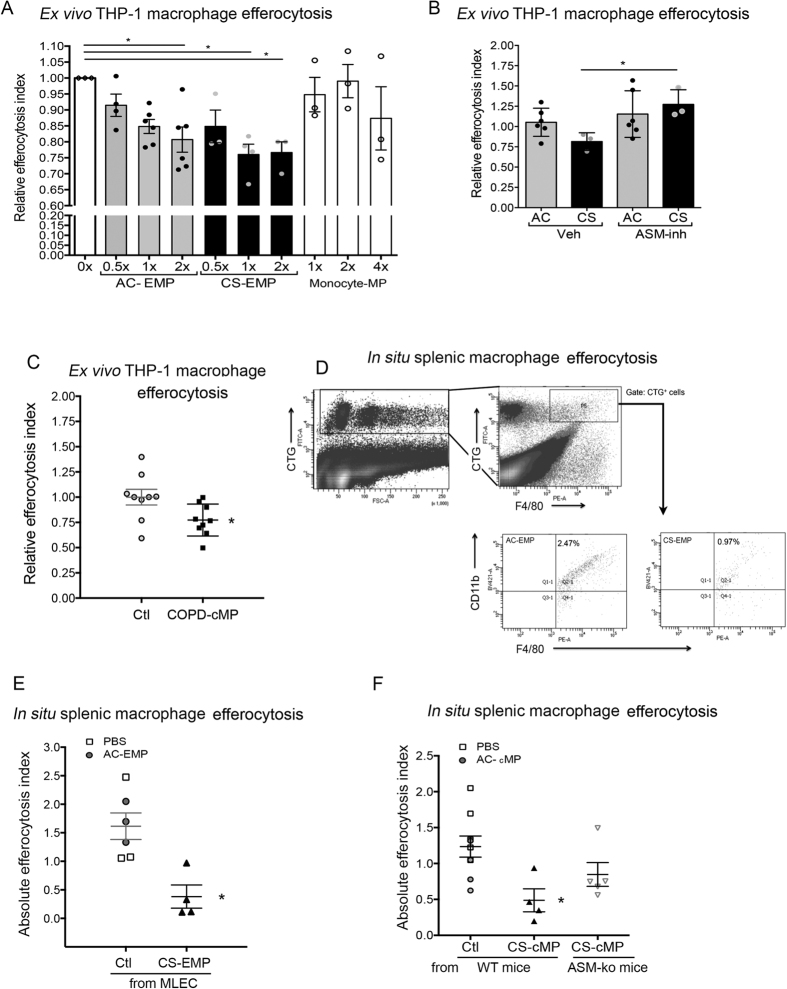
Effect of microparticles on macrophage efferocytosis. (**A**) Relative efferocytosis index (fold vs. EMPs-free) of apoptotic Jurkat cells by THP-1 in the presence of MPs (x indicates fold concentration) released by HLMVECs (exposed AC or CS extract, 5%, 16 h, n = 3–4) or by human peripheral blood monocytes (differentiated with 5 ng/mL PMA, 48 h, n = 3). Mean ± SEM; ANOVA (#p < 0.05 vs. untreated; *p < 0.05 vs. AC-treated cells). (**B)** Relative efferocytosis index (fold vs. EMPs-free) of apoptotic cells in the presence of EMPs (1 x) released by HLMVECs pre-treated with aSMase inhibitor (imipramine; 50*μ*M) prior CS exposure (5%, 16 h, n = 3). Mean ± SEM (ANOVA, *p < 0.05). (**C)** Relative efferocytosis index (fold vs. cMPs-free) of apoptotic cells by THP-1 in the presence of cMPs from COPD individuals (n = 6). Mean ± SEM. ANOVA p < 0.01 (Sidak’s *p < 0.05). **(D–F)** Flow cytometry analysis of engulfed Cell Tracker green (CTG)-labeled apoptotic cells by splenocytes (**D**, left panel) with focus on CTG-gated region with F4/80^+^/CD11b^+^ macrophages that engulfed CTG-labeled apoptotic cells ((**D**) right panels) in spleen homogenates of mice. *In situ* efferocytosis measured as proportion (%) of F4/80^+^/CD11b^+^/CTG^+^ from total F4/80^+^/CD11b^+^ cells (**E,F**) in C57Bl6/J mice injected with vehicle (PBS, 60 μl) or equal numbers of EMPs from either AC- or CS-exposed MLEC ((**E**) 10%, 2 h, Mean ± SEM, t-test; *p < 0.05); or injected with cMPs isolated from equal volume of plasma of wild-type or aSMase (*Smpd1*^−/−^) KO mice exposed to ambient air (AC) or CS ((**F**) 3 h; Mean ± SEM, ANOVA p = 0.02; Dunnetts’s post-hoc *p < 0.05 vs. Ctl).

Since PS-positive MPs can engage specialized receptors for apoptotic cell engulfment, EMPs may compete with other PS-expressing targets such as apoptotic cells, for efferocytotic clearance. To investigate this effect, we used PMA-stimulated human monocytes (THP-1 cell line) co-incubated *ex vivo* with apoptotic cells (UV-irradiated human Jurkat T-cell line) in the presence or absence of EMPs or cMPs. When co-incubated with phagocytic cells and apoptotic cells, all EMPs inhibited efferocytosis, but those shed by CS-exposed HLMVEC had, at the same concentration, a more potent effect (Fig. 4A). To determine if this effect is cell-of-origin-specific, we compared EMPs with monocyte-derived MPs, which failed to significantly inhibit efferocytosis at equal or even 4-fold higher concentrations (Fig. 4A). The EMPs inhibitory effect was related to their abundance, since aSMase inhibition reduced the number of EMPs released by CS and the EMPs collected from imipramine-treated HMLVECs had significantly less inhibitory effect of efferocytosis (Fig. 4B). Similar to EMPs, cMPs isolated from individuals with COPD significantly inhibited apoptotic cells efferocytosis by monocytes (Fig. 4C). To determine if this effect of EMPs is recapitulated *in vivo*, we employed a murine model described by Subramanian *et al.*^29^. In this model, Cell Tracker Green-labeled apoptotic splenocytes are injected intravenously into mice and their *in situ* engulfment by splenic macrophages is quantified using flow cytometry of spleen tissue homogenates (Fig. 4D). Mice injected with EMPs collected from CS-exposed MLEC supernatants had significantly decreased efferocytosis of apoptotic targets by splenic F4/80^+^/CD11b^+^ macrophages, when compared to mice injected with a similar number of EMPs derived from AC-exposed MLEC or with vehicle control (PBS) (Fig. 4D–E). Similarly, mice injected with cMPs isolated from CS-exposed wild type littermate mice had decreased *in situ* splenic macrophage efferocytosis, when compared with mice injected with cMPs isolated from the same volume of plasma from ambient air-exposed mice. Unlike cMPs from wild-type mice, and consistent with the EMPs effects on *ex vivo* efferocytosis, cMPs isolated from CS-exposed aSMase (*Smpd1*^−/−^) mice had a lesser impact on *in situ* splenic macrophages efferocytosis (Fig. 4F).

## Discussion

Our results indicate that acute exposure to soluble components of CS is sufficient to stimulate the release of EMPs consisting of a mixed population of exosomes and membrane particles of much smaller size than apoptotic bodies (3–5 μm). CS-induced EMPs required aSMase activation for their release and encompassed exosomes that carried specific miRNA signatures. In addition, CS-induced EMPs were enriched in ceramides and thus may represent major carriers of these sphingolipids in plasma. The significant inhibitory effect of EMPs on efferocytosis suggests that their clearance may compete with that of apoptotic cells, implicating these membrane structures in conditions associated with excessive apoptosis.

The release of EMPs as a cellular stress response to CS exposure is not surprising, since human umbilical vein endothelial cells release EMPs after activation by inflammatory cytokines, LPS, and oxidative stress^30^; and circulating EMPs are increased during multiple conditions characterized by systemic endothelial cell damage^31^. Our findings indicate that smoking exposure itself releases EMPs, yet further investigations are needed to explain why EMPs were found (both in our small and other much larger cohorts) to be present in higher numbers in the plasma of those smokers with early COPD/emphysema compared to smokers with advanced disease^4^,^5^,^6^. One possibility is that in advanced disease there is loss of pulmonary capillary beds with loss of vascular membrane surface area, or that signals leading to MPs shedding are lost with advanced disease.

Our experimental methods differed from earlier studies by incorporating ultraspeed centrifugation (which eliminates debris and protein aggregates) and depletion of platelet-derived cMPs from plasma for both experimental and clinical samples. Using this methodology, we found that cMPs generated during the acute CS exposure models shared several features with cMPs isolated from subjects with COPD, such as a relative increase in C16-ceramide and functional inhibition of PBMDMs efferocytosis. Given the potential importance of EMPs to clinical phenotyping (for example identifying a subset of at-risk smokers^32^), it will be useful for the field to standardize EMPs and cMPs isolation and quantification.

Our finding of ceramide-containing cMPs provides evidence for alternative carriers of these highly lipophilic molecules in aqueous acellular environments, such as plasma or bronchoalveolar lavage fluid. The existence of bioactive paracellular ceramides has long been suspected^11^,^27^ and attributed to (lipo)protein carriers. It remains to be demonstrated if ceramide-rich cMPs exert ceramide-specific biological effects, which could render them a target for therapy for unwanted outside-in ceramide signaling such as that involved in lung endothelial barrier dysfunction and apoptosis^16^,^25^. The release of ceramide-rich EMPs during CS-exposure by HLMVEC coincided with surface PS and histone expression in EMPs and was dependent on aSMase activation, an enzyme critical for stress-induced apoptosis in multiple cell types. This would be consistent with an apoptotic origin of CS-released EMPs, further supported by reports of EMPs shed in the circulation that parallel endothelial apoptosis^4^, by excessive pulmonary endothelial apoptosis in emphysema^11^, and by expression of apoptosis and endothelial markers on cMPs from smokers and COPD patients^4^,^5^. However, MPs may be released by alternative mechanisms related to inflammatory cell activation^33^,^34^, or to the elimination of the patches of damaged plasma membranes during repair^35^. Indeed, concomitantly with or preceding apoptosis, CS exposure causes endothelial cells inflammatory activation^25^ and induces ER stress^36^. We have not detected the endothelial activation marker CD62E on CS-released EMPs (data not shown), but identified, using NanoSight sizing, a marked release of exosomes. Although the majority of CS-induced EMP stained positive with Annexin V, non-apoptotic MPs such as exosomes have also been shown to externalize PS. Furthermore, only a small proportion of EMPs were the size of membrane particles, which may include the apoptotic vesicles. These results suggest that apoptosis is not the sole or predominant mechanism of CS-induced EMP release. Furthermore, we were unable to detect apoptotic vesicles following acute CS exposures *in vitro* or *in vivo*, and aSMase activation was indispensable to the release of cMPs *in vivo* (where few apoptotic MPs were detected). These findings may indicate that although signaling shared with apoptosis was required, the completion of apoptosis may not be necessary for CS-induced MP release. Indeed, our time-lapse microscopy study showed release of EMPs early during cellular contraction, simultaneous with retraction/resolution of filopodia, without appreciable cell death.

Our results revealed a new function of aSMase, that of release of ceramide-rich EMPs in response to CS exposure. The mechanisms by which aSMase may exert this effect at the plasma membrane, where it breaks down sphingomyelin and enriches the outer membrane leaflet in cholesterol^37^, is *via* promoting membrane fluidity, destabilization, and blebbing^38^, a mechanism which may be instrumental in EMPs release. Although our transgenic approaches implicate the lysosomal form of aSMase (which can translocate to the plasma membrane), they do not rule out soluble aSMase involvement. This notion is supported by our novel finding of significantly increased levels of circulating aSMase in COPD patients plasma. In addition to involvement in generating EMPs, circulating aSMase may breakdown circulating sphingomyelin, which would be consistent with decreased plasma levels of this sphingolipid that we recently described in a large COPD cohort^19^. Several explanations may exist to reconcile the findings of increased plasma aSMase activity in COPD of all severities, while we and others^6^ have found EMPs elevations in smokers and individuals with mild COPD, rather than severe COPD. It is possible that the measurements of aSMase activity are more sensitive than EMPs counts to detect COPD; that EMPs abundance is more closely related to certain disease phenotypes that are not linked to GOLD severity; or that a plateau is reached in aSMase-dependent EMPs release which may be proportional to the lung capillary surface area, (lost with increasing COPD severity).

It is widely recognized that exosomes represent the main transporters of circulating miRNAs and the effects of exosomes in the recipient cells are dependent on the transfer of functional miRNAs^8^,^10^,^39^. Identification and validation of increased hsa-let-7d and hsa-mir-125a, −126, and −191 in CS-induced EMPs are novel and may be important in predicting potential function of exosomes as paracrine effectors in COPD. For example, the release of miRNAs by microvascular pulmonary endothelial cells may modulate the phenotype of distant endothelial cells of large systemic- or pulmonary arteries *via* exosomes uptake. Although we did not interrogate the role of this miR signature in the inhibition of macrophage efferocytosis by EMPs, it is conceivable that miRNAs could modulate this function, given the known roles of let-7d in reducing cell motility^40^,^41^, and that of mir-126 in the cytokine secretion of activated macrophages^42^. Predictions based on the validated targets and recognized functions of the identified miRNAs in our studies include vascular and tissue remodeling, inflammation, and injury repair. For example, let-7d induces cellular apoptosis^43^; mir-126 modulates the repair of damaged endothelium^44^; and circulating mir-191 is increased in smokers^26^ and individuals with pulmonary hypertension^45^. In addition, the relative intracellular loss of respective miRNAs may untowardly affect the function of parent endothelial cells.

We are reporting a new function of CS-released EMPs and cMPs, of hindrance of apoptotic cell clearance (efferocytosis). By impairing reparative macrophage efferocytosis in the systemic or pulmonary microcirculation, circulating EMPs may contribute to the pathogenesis of inflammatory diseases. In principle, this mechanism could affect marginated, but not yet transmigrated monocyte-derived macrophages, which have been recently identified by intravital microscopy in several microcirculatory beds in the kidney, mesentery, and lung^46^,^47^,^48^. Through these effects, EMPs can actively impact local inflammatory responses. The mechanism by which EMPs inhibit efferocytosis may be linked to competition for PS-binding receptors, high ceramide content, or posttranscriptional modifications via miRNA cargo. The inhibition of efferocytosis by high concentrations of AC-derived EMPs together with the weaker effects of CS-derived EMPs on efferocytosis during conditions of aSMase loss of function suggest a competitive effect of EMPs with apoptotic cells for phagocyte engulfment. In addition, critical levels of ceramide in EMPs may hinder efferocytosis, which is consistent with our report that increasing ceramide content in a target lipid vesicle bilayer significantly inhibited macrophages efferocytosis, due to increased membrane stiffness and impaired intercellular membrane fusion^24^. We did detect sphingosine-1 phosphate (S1P) within cMPs (ranging from 0.83 to 1.65 pmoles/cMP in healthy to 1.02 to 2.38 pmoles/cMP in COPD individuals). The differences between these two groups were not statistically significant (data not shown). Therefore, the net result of CS-released cMPs on efferocytosis may be due to higher ceramide content without proportional increase in S1P. This is consistent with our recent report that plasma S1P was not increased in COPD patients enrolled in the COPDGene cohort, and was rather negatively correlated with emphysema severity and risk of COPD acute exacerbation^19^. In contrast to our findings in cMPs, an interesting study identified hepatocyte-derived exosomes that were devoid of S1P, but contained enzymes from the S1P synthetic machinery that were then transferred to injured mouse hepatocytes to increase recipient cell S1P content and restore homeostasis following ischemia/reperfusion injury^49^. Although we did not measure sphingosine kinases in CS-released endothelial exosomes, their miRNA content predicts that upon uptake by recipient monocytes, these miRNAs may actually downregulate S1P synthesis enzymes. Compared to healthy individuals, human cMPs from COPD patients contained higher levels of miR124 and miR101 that are predicted to inhibit sphingosine kinase^50^,^51^. These results may reflect the specificity of exosome structure and function to the cell of origin (hepatocyte vs. endothelial cell) and to the condition in which they are generated (baseline vs. CS-exposure).

Future investigations will have to interrogate whether EMPs directly contribute to COPD pathogenesis, such as lung parenchyma remodeling or airway disease, a hypothesis which is already supported by correlations between EMPs number and lung function decline in murine models^52^ or COPD exacerbations in humans^5^. In addition, EMPs may contribute to all or some of the systemic manifestations of COPD, known as COPD co-morbidities that include cardiovascular events, bone marrow dysfunction, malignancies, impaired cognitive function, and skeletal muscle wasting. A link between EMPs and these manufestations is supported by findings of increased circulating MPs in those with lung cancer cachexia^53^, or in smokers with impaired glucose tolerance, metabolic syndrome and cardiovascular disease^54^.

In conclusion, we revealed that absorbed soluble components of CS directly increase the number of cMPs in mild COPD patients and in murine and cell culture models of CS exposure, by triggering activation of aSMase, an enzyme classically associated with stress-induced apoptosis. Apoptotic and exosomal EMPs released by CS carry ceramides and specific miRNAs in circulation with potential widespread effects in the vasculature of target organs. In addition, EMPs and cMPs are cleared by phagocytes and compete with apoptotic cells for efferocytosis. These novel findings suggest that EMPs may be a useful indicator of the functional state of the endothelium in smokers and may play key roles in inflammation and vascular remodeling changes in response to CS.

## Materials and Methods

Plasma from apparently healthy non-smokers and COPD subjects was obtained after informed consent, using methods that were carried out in accordance with the approved guidelines. All protocols were approved by the IRB at the Indiana University School of Medicine.

Chemicals and reagents were purchased from Sigma-Aldrich (St. Louis, MO), unless otherwise stated.

### Microparticle isolation

#### EMPs

HLMVEC, HPAEC, or MLEC were grown in 100 mm dishes to 98–100% confluence and exposed as indicated in low-serum (2% FBS) media. Cell supernatants were centrifuged twice (350*g*, 8 min, 22^o^C; 1500*g*, 25 min, 22^o^C), to remove floating cells and debris, respectively and underwent a final centrifugation at 100,000*g*, 2 h, 4^o^C to isolate EMPs.

#### Monocyte-derived MPs

Human THP-1 cell line monocytes were plated in 100 mm dishes (2 × 10^6 ^cells/dish) in media with PMA (5 ng/mL; 48 h). Following differentiation, cell supernatant was collected and subjected to similar centrifugation steps as described for EMPs.

#### cMP isolation from human plasma

Healthy non-smokers (n = 8) and COPD (n = 17) subjects were asked to participate in the study under IRB approved clinical protocols. Clinical details are described in the Online Supplement. Blood was drawn from consenting, fasting and resting volunteers using atraumatic butterfly needle system. The first 2–3 mL of blood were discarded and the rest collected in BD Vacutainer Cell Preparation Tubes with sodium citrate. During centrifugation (1600 g, 20 min, 23^o^C) the gradient gel inside the tube separated the mononuclear cells and plasma from the denser blood components. Next the plasma was further centrifuged (1550 g, 25 min, 23^o^C) to obtain platelet-poor plasma. Within minutes the platelet-poor plasma was transferred in BD ultracentrifugation tubes. After ultracentrifugation (100,000g, 4^o^C, 2h) cMPs were used for in various assays: EMPs quantification after CD31 and CD42b immunostaining, ceramides measurements, or efferocytosis assays.

#### Cell culture experiments

Primary mouse lung endothelial cells (MLEC) were kindly provided by Dr. P. Lee (Yale) or isolated in our laboratory from mouse lung using a magnetic cell separation (MACS) procedure from Miltenyi Biotec (Cologne, Germany)^55^. Primary HLMVEC and HPAEC were from Lonza (Walkersville, MD). Culture conditions are detailed in Supplementary Material.

Preparation of soluble cigarette smoke (CS) extract is detailed in Supplementary Material.

#### Time-lapse intravital imaging of cultured cells

HLMVEC were transduced overnight with CellLight^®^ Plasma Membrane-RFP, BacMam2.0 (Molecular Probes). Microscopy was performed as detailed in Supplementary Material. EMP characterization by TEM is detailed in the Supplementary material.

#### EMPs characterization by NanoSight NS300

EMPs isolated by ultracentrifugation were subjected to a PBS wash to eliminate contamination of proteins and other small cell debris before a second ultracentrifugation step. Final EMPs suspension was analyzed using NanoSight NS300, at a rate of 10 μm/min. Three 60-s videos per sample were used to calculate EMPs mode, mean, and total concentration via NTA 3.1 software.

*Microparticle miRNA isolation and analysis* are described in Supplementary Material.

*Ceramide determination* and aSMase assays were performed as previously described^12^ and are detailed in the Online Supplement.

*Animal experiments* were all approved by and conducted in compliance with the guidelines of the Institutional Animal Care and Use Committees of Indiana University School of Medicine and National Jewish Health. Experimental details are provided in Supplementary Material.

*In-vivo and ex-vivo* *efferocytosis assays* are detailed in Supplementary Material. Briefly, mice (C57Bl6, female, 3 month-old) received sequential IV administrations (tail vein) of MLEC-derived EMPs, cMPs, or PBS (60 ul) and, within 15–20 min, fluorescently labeled apoptotic splenocytes (2 × 10^7^). MLEC-derived EMPs were isolated from cultured cell supernatants. To inject the same number of microparticles, EMPs derived from two AC-treated T150 flasks or one CS-treated T150 flask were re-suspended in 60 ul sterile PBS and within minutes administered intravenously into recipient mice. cMPs were isolated from 300 uL plasma of wild type and Smpd1^−/−^ mice exposed to CS for 3 h.To generate apoptotic targets, splenocytes were harvested one-day prior from spleens of healthy C57Bl/6 mice, fluorescently-labeled with Cell Tracker Green (10 ul, 1 mM), and maintained under low serum conditions for 16 h, to undergo spontaneous apoptosis (>85% total cells, detected by Annexin V/7AAD staining and FACS). Recipient animals were sacrificed 3 h after administration of labeled apoptotic splenocytes, spleens were harvested, mechanically digested, and the splenic macrophages/dendritic cell population was identified using membrane antigen immunostaining with F4/80 (1:200, clone CI:A3-1 conjugated with AF647) and CD11b (1:160, clone M1/70 eFlour 450) via flow cytometry (BD-LSR Fortesa cytometer with BD FACSDiva 6.0 software).

*Statistical Analysis* was performed with Prism (GraphPad Software, San Diego, CA), using unpaired Student t-test or ANOVA, as appropriate. When ANOVA was utilized, post-hoc inter-group comparisons were analyzed for statistical significant differences using either Tukey’s (all groups compared to each other), Dunnet’s (groups compared to a control group), or Sidak’s (comparisons of only select pairs of groups) methodology, as appropriate. Statistical significance was accepted at p < 0.05.

## Additional Information

**How to cite this article**: Serban, K. A. *et al.* Structural and functional characterization of endothelial microparticles released by cigarette smoke. *Sci. Rep.* **6**, 31596; doi: 10.1038/srep31596 (2016).

## Supplementary Material

Supplementary Video 1

Supplementary Video 2

Supplementary Video 3

Supplementary Information

## Figures and Tables

**Table 1:** miRNAs most increased by CS exposure in both mouse cMPs and human EMPs.

Probeset ID	Fold CS/AC (EMP)	Fold CS/AC (cMP)	*Meta analysis p-value (CS*/*AC)*	FDR (RM-ANOVA)	Sequence	Putative function
let-7d_st	2.14	1.6	*8.9E-03*	9.8E-01	AGAGGUAGUAGGUUGCAUAGUU	Allergic airway inflammation/ asthma^56^; lung fibroblast EMT^57^; cell cycle arrest, apoptosis^58^
miR-126_st	1.11	3.74	*2.0E-05*	3.5E-03	UCGUACCGUGAGUAAUAAUGCG	Angiogenesis, endothelial activation^59^,^60^; VSM turnover^61^,^62^; EPC proliferation/differentiation^63^
miR-125a-5p_st	1.46	2.42	*5.5E-03*	1.9E-01	UCCCUGAGACCCUUUAACCUGUGA	Angiogenesis^64^; NSCLC progression 65; macrophage differentiation^66^
miR-26a_st	1.15	1.96	*1.1E-07*	4.7E-05	UUCAAGUAAUCCAGGAUAGGCU	NSCLC progression, metastasis^67^
miR-99a_st	1.10	2.01	*4.6E-05*	7.2E-03	AACCCGUAGAUCCGAUCUUGUG	Inhibition of cancer cell invasiveness and proliferation^68^
let-7c_st	1.60	1.34	*1.1E-6*	3.3E-04	UGAGGUAGUAGGUUGUAUGGUU	Inhibition of migration, proliferation, and invasion of NSCLC^69^
miR-33b-star_st	1.27	1.59	*3.7E-03*	1.5E-01	CAGUGCCUCGGCAGUGCAGCCC	Lipid metabolism^70^
let-7a_st	1.19	1.56	*5.6E-07*	1.9E-04	UGAGGUAGUAGGUUGUAUAGUU	Inhibition of cancer cell proliferation^71^
miR-22_st	1.35	1.35	*6.5E-08*	3.8E-05	AAGCUGCCAGUUGAAGAACUGU	Inhibition of cancer cell proliferation^72^; apoptosis^73^

Candidates identified by meta analysis (p < 0.01). Abbreviations: EPC: endothelial progenitor cells; NSCLC: non-small cell lung cancer.
