# Supplementary material for: Structural and functional characterization of endothelial microparticles released by cigarette smoke
Source: Sci Rep. 2016 Aug 17;6:31596. doi: 10.1038/srep31596 (PMC4987682; doi:10.1038/srep31596)
Supplement: Supplementary Information [file srep31596-s4.doc]

**Supplementary material**

**Structural and functional characterization of endothelial microparticles released by cigarette smoke**

Karina A. Serban^1,2^*, Samin Rezania^1^*, Daniela N. Petrusca^1^*, Christophe Poirier^1&^, Danting Cao^2,3^, Matthew J. Justice^2,3^, Milan Patel^1^, Irina Tsvetkova^5^, Krzysztof Kamocki^3^, Andrew Mikosz^2^, Kelly S. Schweitzer^1,2^, Sean Jacobson^5^ , Angelo Cardoso^4^, Nadia Carlesso^4^, Walter C. Hubbard^6^, Katerina Kechris^2^, Bogdan Dragnea^5^, Evgeny V. Berdyshev ^2,7^, Jeanette McClintock^3^, and Irina Petrache^1, 2, 8#^

**Supplementary methods**

Chemicals and reagents: Ceramide with C16:0 fatty acyl chain (conjugated with polyethylene glycol 2000) was purchased from Avanti Polar Lipids (Alabaster, AL). Imipramine was from Calbiochem, San Diego, CA. Ceramide mAb (200 µg/ml) were from Enzo Life Science (Farmingdale, NY). The TACS® Annexin V-FITC Kit was from Trevigen (Gaithersburg, MD). Phorbol myristate acetate (PMA) was from Abcam (Cambridge, MA).

Cell culture experiments: Briefly, MLECs were isolated as follows: lung cell suspensions were incubated with microbeads conjugated with CD146 antibody. CD146-positive cells (endothelial cells) were isolated using magnetic field selection and were cultured on gelatin-coated tissue culture plates in high-glucose Dulbecco's Modified Eagle Media (DMEM; Hercules, CA) supplemented with 20% Fetal Bovine Serum (FBS), L-glutamine, and 1% penicillin and streptomycin. HLMVEC and HPAEC were from Lonza. HLMVEC were maintained in complete culture medium consisting of EGM-2MV supplemented with their specific SingleQuots® (Lonza, Walkersville, MD). Cultured cells were maintained at 37^o^C with 5% CO2 and were used for experiments between passages 7 to 18 for MLEC and 3 to 9 for HLMVEC cells at 70-80% confluence.

Preparation of soluble cigarette smoke (CS) extract: Filtered research grade cigarettes (3R4F) from the Kentucky Tobacco Research and Development Center (University of Kentucky, Lexington, KY) were used for preparing an aqueous CS extract, as described ^1^. CS (100%) was prepared by bubbling smoke from 2 cigarettes into 20 ml of PBS at a rate of 1 cigarette/min to 0.5 cm from the filter, followed by pH adjustment to 7.4 and 0.2 µm filtration. A similar procedure was followed for the control air extract (AC) preparation, where ambient air was bubbled into the cell culture medium. Treatments were performed with the indicated CS extract concentration (vol:vol).

Microparticle counting by flow cytometry: EMPs pellets were resuspended in PBS and stained for lipids with Nile Red (1:100). EMPs quantification was normalized by counting the total number of events for 240 seconds for each sample expressed by volume of cell culture supernatant. Events were acquired on a Beckman Coulter Cytomics FC500 cytofluorimeter with CXP software version 2 (Beckman Coulter, Fullerton, CA).

Microparticle immunostaining

*MLEC-derived MPs immunostaining:* EMPs were re-suspended in FACS buffer (PBS; 2% BSA; 0.01% sodium azide), incubated with Fc block (BD Bioscience), and stained in FACS buffer with: PE anti-mouse CD63 antibody, PE Rat IgG2a, κ isotype control (Biolegend); anti-histone H3 primary antibody and Cy3-conjugated goat anti-mouse (Abcam; Cambridge, MA) secondary antibody, using rabbit IgG antibody (Abcam) control; PE anti-mouse CD62 antibody (BD Pharmingen^TM^) and FITC anti-mouse CD31 (BD Pharmingen^TM^).

*Human circulating MPs immunostaining:* cMPs pellets were suspended in FACS buffer and incubated with FITC anti-human CD31 (1:25, Sigma-Aldrich) or PE-anti human CD42b (1:20; Thermo Scientific, IL) and respective isotype controls.

*MPs Annexin V staining:* EMPs or cMPs were resuspended in 50 µl binding buffer and then incubated with 5µl PE Annexin V (1:10, BD Biosciences, San Diego, CA), followed by flow cytometry quantification.

cMP isolation from **human plasma**: Healthy non-smokers (n=8) and COPD (n=17) subjects were asked to participate in the study under IRB approved clinical protocols. *Inclusion criteria*: males and females, age 18 or older; healthy controls never-smokers by history; and mild to severe COPD defined by GOLD 2011 guidelines. *Exclusion criteria*: pregnancy; current active infection or acute illness; current alcohol or drug abuse; malignancy (past 5 years); anemia (to protect from unnecessary phlebotomy); and systemic conditions that may affect EMPs: interstitial lung disease, pulmonary hypertension, coronary artery disease or heart failure, stroke, peripheral vascular disease, and systemic autoimmune disease. Consenting fasting volunteers had a blood draw following a rest period of at least 30min. An atraumatic butterfly needle system was used for sample collection in all individuals. Following venipuncture, the first 2-3 mL of blood were discarded. Blood samples were collected in two 8 ml BD Vacutainer Cell Preparation Tubes with sodium citrate. The collected blood was mixed with anticoagulant by gentle inversion 8-10 times. All samples were kept at room temperature and further processed within 30-40 minutes after collection. The BD Vacutainer CPT system contains sodium citrate anticoagulant and blood separation media composed of a thixotropic polyester gel and a FICOLL Hypaque solution. During centrifugation (1600g, 20 min, 23 ^o^C) the gel portion of the medium forms a barrier separating the mononuclear cells and plasma from the denser blood components. Next the plasma underwent further centrifugations (1550g, 25 min; 100000g, 2h) to obtain cMPs pellets.

**Time-lapse intravital imaging of cultured cells :** HLMVEC were transduced overnight with CellLight® Plasma Membrane-RFP, BacMam 2.0 (Molecular Probes®) according to the manufacturer’s instructions. Cells were treated with different concentrations of CS extract (2% or 5%) or the respective air controls and visualized by time-lapse fluorescence video microscopy every 2 min for 1h. Images were acquired using a Perkin-Elmer spinning disk confocal microscope system mounted on a Nikon TE 2000 U inverted microscope, using Nikon ×100 NA 1.4 oil immersion plan apochromatic objective. Fluorescent images of the cells were taken by using an appropriate combination of excitation/emission (555/584 nm) filters. The system was equipped with an automatic stage-positioning system used to record the positions of several cells selected across each plate, allowing multiple cells to be studied in each experiment. Images were analyzed using NIS Elements AR 4.3 Software.

**EMP characterization by TEM:** Two types of sample preparations for electron microscopy were used. The first was for studying of unstained samples and the second used negative staining by phosphotungstic acid. Specimens were prepared by placing a 10 μL drop of stock sample onto a carbon-coated 400 mesh copper grid. After 10 min incubation at room temperature, the excess solution on the grid was removed with filter paper. Unstained samples were left to dry at room temperature for 30 minutes before imaging. For stained samples, a 10 μL drop of 3% phosphotungstic acid was placed on the blotted sample and left for 10 min. Excess solution was then removed by blotting with filter paper and samples were left to dry at room temperature for 30 min in a desiccator before imaging. Images were acquired at an accelerating voltage of 80 kV on a JEOL JEM1010 transmission electron microscope equipped with Gatan UltraScan 4000 CCD camera.

**Microparticles miRNA isolation and expression analysis:** Following lysis with Quiazol Lysis Reagent and chloroform and ethanol precipitation, total RNA was extracted using the miRNeasy Mini Kit (Quiagen) and RNeasy Mini spin columns. Small RNA samples were labeled using the Affymetrix FlashTag HSR kit. The labeled samples were hybridized to Affymetrix GeneChip® miRNA 3.0 arrays. They were stained and washed using the standard miRNA protocol. Affymetrix GeneChip Command Console Software (AGCC) was used to scan the arrays and generate CEL files. CEL files were imported into Partek Genomics Suite for analysis. RMA (robust multi-array average) signals were generated for the probe sets using the RMA background correction, Quantile normalization and summarization by Median Polish ^2^. Summarized signals for each probe set were log_2_ transformed and used for Principal Components Analysis to determine if there were any outlier arrays, and no outliers were detected. Student’s t test or one-way ANOVA was performed as appropriate, using the log base 2 transformation of the expression levels. Untransformed RMA signals were used for fold change calculations. To perform the meta-analysis, for each human probeset (excluding stem loops), all mouse probesets with the same miRNA annotation were matched. If there were multiple probesets matching in the mouse array, repeated-measures ANOVA was performed. Then, the p-values for the human and mouse microarray results were combined using the Stouffer Z-score ^3^. False discovery rate (FDR) was calculated using the Storey method ^4^.

Ceramide determination was performed as previously described ^5^.. Following treatments, either fresh or flash-frozen EMPs were suspended in methanol or cells were collected in methanol following culture media removal and washing with PBS. Lipids were extracted utilizing a modified Bligh and Dyer method and total lipid phosphorus (Pi) content of each lipid extract was measured by NH4-molybdate labeling, as previously described ^5^. Sphingolipid analyses were performed via combined liquid chromatography-tandem mass spectrometry using AB-Sciex 5500 QTRAP hybrid triple quadrupole ion trap mass spectrometer (Foster City, CA) interfaced with an Agilent 1200 series liquid chromatograph (Agilent Technologies, Wilmington, DE). Ceramide analytes were ionized *via* positive ion electrospray ionization. Elution of the ceramides was detected by multiple reaction-monitoring characteristic for 14:0, 16:0, 18:0,18:1, 20:0, 24:0 and 24:1 ceramides. C17:0-ceramide was employed as internal standard. All ceramide measurements were normalized by lipid Pi.

Acid sphingomyelinase assay: Murine lung tissue and cultured cell lines were harvested in specific buffers, as described ^6^. ASMase activity was measured with Amplex Red Sphingomyelinase Assay Kit (Molecular Probes, Eugene, OR) following manufacturer’s protocol, using fluorescence Spectra Max microplate reader (Molecular Devices, Sunnyvale, CA). Hydrogen peroxide and purified sphingomyelinase were used as positive controls.

**Ex-vivo efferocytosis** assessment and quantification were performed as previously described^7^**.** Briefly, human monocytes (THP-1 cell line) were accommodated in culture media supplemented with PMA (5ng/mL, 24 h). Apoptotic targets were obtained from Jurkat cells labeled with Cell Tracker Orange (Invitrogen, Grand Island, NY; 0.5 mM) and exposed to UV radiation (30,000 μJ/cm^2^) using a HL-2000 HybriLinker, followed by incubation for 3.5 h at 37^o^C, 5% CO2 in serum-free media. Labeled apoptotic Jurkat cells were then co-incubated (1 h, 37^o^C) with THP-1 monocytes (5:1) in the presence or absence of MPs. Adherent cells were collected and the extracellular fluorescence (of membrane-bound but non-engulfed labeled apoptotic cells or bodies) was quenched with trypan blue (Sigma; 1000μl; 0.04% in PBS). Efferocytosis was quantified by FACS using Cytomics FC500 cytofluorimeter with CXP software (Beckman Coulter, Fullerton, CA) and expressed as efferocytosis index (relative to control condition for individual experiments).

**Animal Studies:** All animal studies were conducted in compliance with the Institutional Animal Care and Use Committee guidelines of Indiana University and National Jewish Health. C57BL/6J mice, male, 7 weeks old, were from Jackson Laboratory (Bar Harbor, ME). Mice carrying a deletion in the Smpd1 gene (Smpd1^-/-^) were from Dr. Schuchman (Mount Sinai Hospital, NY) ^8^.

Transgenic mice carrying at the X-linked *Hprt* locus the mutation Hprt^tm2.1(CAG-Smpd1)Geno^ ^9^, had a stop cassette flanked by 2 LoxP sites is inserted between the ubiquitous CAG promoter and the mouse *Smpd1* cDNA. To induce aSMase over-expression in endothelium, we used the Tg(Tek-cre/ERT2)1Arnd mouse ^10^, which expresses under the endothelium-specific Tek promoter a modified CRE DNA recombinase activated by tamoxifen ^11^. Tamoxifen (20μg/mL) or vehicle (10% ethanol: 90% sunflower seed oil, vol:vol) were administered daily by gavage (200μL).

**Cigarette smoking exposure of mice** was performed as previously described, using a whole-body exposure model to Kentucky research cigarettes for 1h, 3h, or 24h, using a Teague 10E whole body exposure apparatus (Teague Enterprise, CA)^12^. Research-grade cigarettes (1R3F, Kentucky Tobacco Research and Development Center, Lexington, KY, USA) were smoked at a rate that achieved 90-120 ng/m^3^ microparticles in the exposure chamber, and optimal CO parameters (average 350 ppm) were set at the chamber setup by the manufacturer. Immediately following exposure, mice were anesthetized by isoflurane inhalation. Blood was collected from the right ventricle in a 1 ml syringe containing 0.2 mL of 3.8% sodium citrate and transferred in a 15 ml tube containing 2.8 mL of 3.8% sodium citrate and stored on ice. cMPs were isolated as previously described for cell culture, except that first centrifugation was performed at 1,550 g for 25 min at room temperature, to remove platelets.

**In-vivo efferocytosis** assessment and quantification were performed as previously described ^13^. We measured efferocytosis index using FACS in splenic macrophages and dendritic cells of C57BL/6 mice injected with circulating microparticles (cMPs), MLEC-derived microparticles (EMPs), or PBS control 30min prior to apoptotic splenocytes IV administration.

*Circulating or endothelial cell-derived MP isolation:*

Mouse lung endothelial cells (MLECs, p4-8) were maintained in culture. For endothelial microparticle (EMP) isolation MLEC were plated in T150 flask and cultured to ~90-100% confluence. Following treatment with 10% AC or CS extract for 16h the supernatant is collected for EMP isolation and centrifuged at 300g then 1500g to remove necrotic cells and cell debris. EMPs pellet is isolated by ultracentrifugation (100,000g, 2h, 4C) and immediately administered i.v. into the recipient mouse.

Circulating MPs (cMPs) were isolated from 300ul plasma from C57Bl/6 (WT) or Smpd1^-/-^ mice exposed to either AC or CS for 3 hours (whole body second-hand cigarette smoke exposure). The mice were euthanized by isoflurane and bilateral pneumothorax and the whole blood was collected by cardiac puncture. Circulating MPs pellet was obtained by a series of centrifugations (1500g, 25min, RT) and ultracentrifugation (100, 000g, 2h, 4C). The cMPs pellet was resuspended in sterile PBS (60uL) and immediately administered i.v. into the recipient mouse.

EMPs or cMPs were injected i.v in the tail vein 30min prior to labeled apoptotic splenocytes administration.

*Labeled and apoptotic splenocytes:*

One day prior to efferocytosis experiment spleens from healthy, untreated C57Bl/6 mice were harvested and digested to isolate individual splenocytes. The excised spleen was sliced into small pieces (mechanical digestion). The suspension was passed through a strainer attached to a 50-mL conical tube. Cells are washed with excess PBS and then centrifuged (300g, 5min, RT). If needed, the cells were treated with warm 2mL RBC lysing solution for 2 min at RT, and then washed with 30 mL of PBS and centrifuged at 300g, 5 min, RT. The cells were re-suspended in RPMI media containing 2% FBS, 2mM glutamine, 1mM sodium pyruvate, 1% nonessential amino acids, 1% penicillin-streptomycin. Before cultured in a 37°C incubator with 5% CO2 for 16h the splenocytes were labeled with Cell Tracker Green (10μl/10 mL media). In a pilot experiment, the Annexin V/PI immunostaining of unlabeled splenocytes confirmed >85% apoptosis as determined by FACS.

The recipient animals were sacrificed 3 hours after the labeled, apoptotic splenocytes administration. Spleens were harvested, mechanically digested, and the splenic macrophages/dendritic cell population was identified using membrane antigen immunostaining with F4/80 (1:200, clone CI:A3-1 conjugated with AF647) and CD11b (1:160, clone M1/70 eFlour 450) via flow cytometry (BD-LSR Fortesa cytometer with BD FACSDiva 6.0 software).

**Supplementary Bibliography**

1. Schweitzer, K.S., Hatoum, H., Brown, M.B., Gupta, M., Justice, M.J., Beteck, B., Van Demark, M., Gu, Y., Presson, R.G., Jr., Hubbard, W.C. & Petrache, I. Mechanisms of lung endothelial barrier disruption induced by cigarette smoke: role of oxidative stress and ceramides. *American journal of physiology. Lung cellular and molecular physiology* **301**, L836-846 (2011).

2. Irizarry, R.A., Bolstad, B.M., Collin, F., Cope, L.M., Hobbs, B. & Speed, T.P. Summaries of Affymetrix GeneChip probe level data. *Nucleic Acids Res* **31**, e15 (2003).

3. Stouffer, S.A. *The American soldier*, (Princeton University Press, Princeton,, 1949).

4. Storey, J.D. & Tibshirani, R. Statistical significance for genomewide studies. *Proceedings of the National Academy of Sciences of the United States of America* **100**, 9440-9445 (2003).

5. Petrusca, D.N., Van Demark, M., Gu, Y., Justice, M.J., Rogozea, A., Hubbard, W.C. & Petrache, I. Smoking exposure induces human lung endothelial cell adaptation to apoptotic stress. *American journal of respiratory cell and molecular biology* **50**, 513-525 (2014).

6. Petrache, I., Natarajan, V., Zhen, L., Medler, T.R., Richter, A.T., Cho, C., Hubbard, W.C., Berdyshev, E.V. & Tuder, R.M. Ceramide upregulation causes pulmonary cell apoptosis and emphysema-like disease in mice. *Nature medicine* **11**, 491-498 (2005).

7. McCaslin, C.A., Petrusca, D.N., Poirier, C., Serban, K.A., Anderson, G.G. & Petrache, I. Impact of alginate-producing Pseudomonas aeruginosa on alveolar macrophage apoptotic cell clearance. *Journal of cystic fibrosis : official journal of the European Cystic Fibrosis Society* **14**, 70-77 (2015).

8. Horinouchi, K., Erlich, S., Perl, D.P., Ferlinz, K., Bisgaier, C.L., Sandhoff, K., Desnick, R.J., Stewart, C.L. & Schuchman, E.H. Acid sphingomyelinase deficient mice: a model of types A and B Niemann-Pick disease. *Nature genetics* **10**, 288-293 (1995).

9. Muhle, C., Huttner, H.B., Walter, S., Reichel, M., Canneva, F., Lewczuk, P., Gulbins, E. & Kornhuber, J. Characterization of acid sphingomyelinase activity in human cerebrospinal fluid. *PloS one* **8**, e62912 (2013).

10. Forde, A., Constien, R., Grone, H.J., Hammerling, G. & Arnold, B. Temporal Cre-mediated recombination exclusively in endothelial cells using Tie2 regulatory elements. *Genesis* **33**, 191-197 (2002).

11. Metzger, D., Clifford, J., Chiba, H. & Chambon, P. Conditional site-specific recombination in mammalian cells using a ligand-dependent chimeric Cre recombinase. *Proceedings of the National Academy of Sciences of the United States of America* **92**, 6991-6995 (1995).

12. Clauss, M., Voswinckel, R., Rajashekhar, G., Sigua, N.L., Fehrenbach, H., Rush, N.I., Schweitzer, K.S., Yildirim, A.O., Kamocki, K., Fisher, A.J., Gu, Y., Safadi, B., Nikam, S., Hubbard, W.C., Tuder, R.M., Twigg, H.L., 3rd, Presson, R.G., Sethi, S. & Petrache, I. Lung endothelial monocyte-activating protein 2 is a mediator of cigarette smoke-induced emphysema in mice. *The Journal of clinical investigation* **121**, 2470-2479 (2011).

13. Kumar, M., Ahmad, T., Sharma, A., Mabalirajan, U., Kulshreshtha, A., Agrawal, A. & Ghosh, B. Let-7 microRNA-mediated regulation of IL-13 and allergic airway inflammation. *The Journal of allergy and clinical immunology* **128**, 1077-1085 e1071-1010 (2011).

14. Pandit, K.V., Corcoran, D., Yousef, H., Yarlagadda, M., Tzouvelekis, A., Gibson, K.F., Konishi, K., Yousem, S.A., Singh, M., Handley, D., Richards, T., Selman, M., Watkins, S.C., Pardo, A., Ben-Yehudah, A., Bouros, D., Eickelberg, O., Ray, P., Benos, P.V. & Kaminski, N. Inhibition and role of let-7d in idiopathic pulmonary fibrosis. *American journal of respiratory and critical care medicine* **182**, 220-229 (2010).

15. Kishore, S., Jaskiewicz, L., Burger, L., Hausser, J., Khorshid, M. & Zavolan, M. A quantitative analysis of CLIP methods for identifying binding sites of RNA-binding proteins. *Nature methods* **8**, 559-564 (2011).

16. Yan, B. & Zhao, J.L. miR-1228 prevents cellular apoptosis through targeting of MOAP1 protein. *Apoptosis : an international journal on programmed cell death* **17**, 717-724 (2012).

17. Lena, A.M., Mancini, M., Rivetti di Val Cervo, P., Saintigny, G., Mahe, C., Melino, G. & Candi, E. MicroRNA-191 triggers keratinocytes senescence by SATB1 and CDK6 downregulation. *Biochemical and biophysical research communications* **423**, 509-514 (2012).

18. Jima, D.D., Zhang, J., Jacobs, C., Richards, K.L., Dunphy, C.H., Choi, W.W., Au, W.Y., Srivastava, G., Czader, M.B., Rizzieri, D.A., Lagoo, A.S., Lugar, P.L., Mann, K.P., Flowers, C.R., Bernal-Mizrachi, L., Naresh, K.N., Evens, A.M., Gordon, L.I., Luftig, M., Friedman, D.R., Weinberg, J.B., Thompson, M.A., Gill, J.I., Liu, Q., How, T., Grubor, V., Gao, Y., Patel, A., Wu, H., Zhu, J., Blobe, G.C., Lipsky, P.E., Chadburn, A., Dave, S.S. & Hematologic Malignancies Research, C. Deep sequencing of the small RNA transcriptome of normal and malignant human B cells identifies hundreds of novel microRNAs. *Blood* **116**, e118-127 (2010).

19. Ge, J., Chen, Z., Li, R., Lu, T. & Xiao, G. Upregulation of microRNA-196a and microRNA-196b cooperatively correlate with aggressive progression and unfavorable prognosis in patients with colorectal cancer. *Cancer cell international* **14**, 128 (2014).

20. Echeverry, N., Bachmann, D., Ke, F., Strasser, A., Simon, H.U. & Kaufmann, T. Intracellular localization of the BCL-2 family member BOK and functional implications. *Cell death and differentiation* **20**, 785-799 (2013).

21. Howitt, J., Low, L.H., Putz, U., Doan, A., Lackovic, J., Goh, C.P., Gunnersen, J., Silke, J. & Tan, S.S. Ndfip1 represses cell proliferation by controlling Pten localization and signaling specificity. *Journal of molecular cell biology* **7**, 119-131 (2015).

22. Mund, T. & Pelham, H.R. Regulation of PTEN/Akt and MAP kinase signaling pathways by the ubiquitin ligase activators Ndfip1 and Ndfip2. *Proceedings of the National Academy of Sciences of the United States of America* **107**, 11429-11434 (2010).

23. Hu, R., Liu, W., Li, H., Yang, L., Chen, C., Xia, Z.Y., Guo, L.J., Xie, H., Zhou, H.D., Wu, X.P. & Luo, X.H. A Runx2/miR-3960/miR-2861 regulatory feedback loop during mouse osteoblast differentiation. *The Journal of biological chemistry* **286**, 12328-12339 (2011).

24. Kozomara, A. & Griffiths-Jones, S. miRBase: annotating high confidence microRNAs using deep sequencing data. *Nucleic Acids Res* **42**, D68-73 (2014).

25. Hsu, S.D., Tseng, Y.T., Shrestha, S., Lin, Y.L., Khaleel, A., Chou, C.H., Chu, C.F., Huang, H.Y., Lin, C.M., Ho, S.Y., Jian, T.Y., Lin, F.M., Chang, T.H., Weng, S.L., Liao, K.W., Liao, I.E., Liu, C.C. & Huang, H.D. miRTarBase update 2014: an information resource for experimentally validated miRNA-target interactions. *Nucleic Acids Res* **42**, D78-85 (2014).

26. Vlachos, I.S., Paraskevopoulou, M.D., Karagkouni, D., Georgakilas, G., Vergoulis, T., Kanellos, I., Anastasopoulos, I.L., Maniou, S., Karathanou, K., Kalfakakou, D., Fevgas, A., Dalamagas, T. & Hatzigeorgiou, A.G. DIANA-TarBase v7.0: indexing more than half a million experimentally supported miRNA:mRNA interactions. *Nucleic Acids Res* **43**, D153-159 (2015).

27. Quintavalle, M., Elia, L., Condorelli, G. & Courtneidge, S.A. MicroRNA control of podosome formation in vascular smooth muscle cells in vivo and in vitro. *The Journal of cell biology* **189**, 13-22 (2010).

28. Fish, J.E., Santoro, M.M., Morton, S.U., Yu, S., Yeh, R.F., Wythe, J.D., Ivey, K.N., Bruneau, B.G., Stainier, D.Y. & Srivastava, D. miR-126 regulates angiogenic signaling and vascular integrity. *Developmental cell* **15**, 272-284 (2008).

29. Wang, S., Aurora, A.B., Johnson, B.A., Qi, X., McAnally, J., Hill, J.A., Richardson, J.A., Bassel-Duby, R. & Olson, E.N. The endothelial-specific microRNA miR-126 governs vascular integrity and angiogenesis. *Developmental cell* **15**, 261-271 (2008).

30. Hergenreider, E., Heydt, S., Treguer, K., Boettger, T., Horrevoets, A.J., Zeiher, A.M., Scheffer, M.P., Frangakis, A.S., Yin, X., Mayr, M., Braun, T., Urbich, C., Boon, R.A. & Dimmeler, S. Atheroprotective communication between endothelial cells and smooth muscle cells through miRNAs. *Nature cell biology* **14**, 249-256 (2012).

31. Zhou, J., Li, Y.S., Nguyen, P., Wang, K.C., Weiss, A., Kuo, Y.C., Chiu, J.J., Shyy, J.Y. & Chien, S. Regulation of vascular smooth muscle cell turnover by endothelial cell-secreted microRNA-126: role of shear stress. *Circulation research* **113**, 40-51 (2013).

32. Meng, S., Cao, J.T., Zhang, B., Zhou, Q., Shen, C.X. & Wang, C.Q. Downregulation of microRNA-126 in endothelial progenitor cells from diabetes patients, impairs their functional properties, via target gene Spred-1. *J Mol Cell Cardiol* **53**, 64-72 (2012).

33. Li, P., Liu, Y., Yi, B., Wang, G., You, X., Zhao, X., Summer, R., Qin, Y. & Sun, J. MicroRNA-638 is highly expressed in human vascular smooth muscle cells and inhibits PDGF-BB-induced cell proliferation and migration through targeting orphan nuclear receptor NOR1. *Cardiovasc Res* **99**, 185-193 (2013).

34. Carrer, M., Liu, N., Grueter, C.E., Williams, A.H., Frisard, M.I., Hulver, M.W., Bassel-Duby, R. & Olson, E.N. Control of mitochondrial metabolism and systemic energy homeostasis by microRNAs 378 and 378*. *Proceedings of the National Academy of Sciences of the United States of America* **109**, 15330-15335 (2012).

35. Song, G., Sharma, A.D., Roll, G.R., Ng, R., Lee, A.Y., Blelloch, R.H., Frandsen, N.M. & Willenbring, H. MicroRNAs control hepatocyte proliferation during liver regeneration. *Hepatology* **51**, 1735-1743 (2010).

36. Spierings, D.C., McGoldrick, D., Hamilton-Easton, A.M., Neale, G., Murchison, E.P., Hannon, G.J., Green, D.R. & Withoff, S. Ordered progression of stage-specific miRNA profiles in the mouse B2 B-cell lineage. *Blood* **117**, 5340-5349 (2011).

37. Dejean, E., Renalier, M.H., Foisseau, M., Agirre, X., Joseph, N., de Paiva, G.R., Al Saati, T., Soulier, J., Desjobert, C., Lamant, L., Prosper, F., Felsher, D.W., Cavaille, J., Prats, H., Delsol, G., Giuriato, S. & Meggetto, F. Hypoxia-microRNA-16 downregulation induces VEGF expression in anaplastic lymphoma kinase (ALK)-positive anaplastic large-cell lymphomas. *Leukemia* **25**, 1882-1890 (2011).

38. Griffiths-Jones, S. The microRNA Registry. *Nucleic Acids Res* **32**, D109-111 (2004).

39. Griffiths-Jones, S., Grocock, R.J., van Dongen, S., Bateman, A. & Enright, A.J. miRBase: microRNA sequences, targets and gene nomenclature. *Nucleic Acids Res* **34**, D140-144 (2006).

40. Griffiths-Jones, S., Saini, H.K., van Dongen, S. & Enright, A.J. miRBase: tools for microRNA genomics. *Nucleic Acids Res* **36**, D154-158 (2008).

41. Kozomara, A. & Griffiths-Jones, S. miRBase: integrating microRNA annotation and deep-sequencing data. *Nucleic Acids Res* **39**, D152-157 (2011).

**Supplementary tables and figures**

**Supplementary Table 1.** **Patient demographic, clinical, and functional characteristics.**

|  | **Healthy** | **COPD** |
| --- | --- | --- |
| Patients | 8 | 17 |
| Gender (F, %) | 6 (75) | 10 (58.8) |
| Race (W, %) | 7 (87.5) | 14 (82.4) |
| Age (years) | 37.4 ± 7.2 | 56.6 ± 10.4* |
| Smoking status (smokers, %) | 0 | 8 (47) |
| Smoking history (PY) | 1.9 ± 4.9 | 37 ± 26.6* |
| SpO_2_ (%) | 98.9 ± 1.1 | 93.9 ± 4.4* |
| GOLD stage 0 | NA | 3 (17.6) |
| GOLD stage 1-2 | NA | 7 (41.2) |
| GOLD stage III | NA | 1 (5.8) |
| GOLD stage IV | NA | 6 (35.3) |
| Exacerbations ≥ 2/ yr (%) | NA | 8 (47) |
| Comorbidity index | 0 | 0.8 ± 0.9 |
| FEV_1_ (%predicted) | NA | 54.2 ± 23.3 |
| DLCO (%predicted) | NA | 51.4 ± 20.6 |

Data are presented as mean ± SD or number (%), unless otherwise stated; *p<0.05 (Student’s t-test). Comorbidity index was calculated by assigning 1 point to each of the following: coronary artery disease, heart failure, essential hypertension, diabetes mellitus, carotid artery disease, and history of cancer (>5 years ago). Abbreviations: PY: pack-year; SpO_2_: peripheral oxygen saturation; GOLD: the Global initiative for chronic Obstructive Lung Disease; FEV_1_: forced expiratory volume in 1 s; DLCO: single-breath diffusion capacity of the lung for carbon monoxide.

Supplementary Table 2. miRNA detected in EMPs from HLMVEC and modulation by CS

| Probeset ID | Mean (AC) | Mean (CS) | Fold (CS/AC) | *p-value* CS vs. AC; FDR 0.8 | Major confirmed (or highly predicted) targets | Putative function |
| --- | --- | --- | --- | --- | --- | --- |
| miRNAs, most altered by CS exposure (p<0.01) | | | | | |  |
| hsa-let-7d_st | 1.84 | 2.93 | 2.14 | *0.003* | IL13, HMGA2, CDC25A, GAB2, SMAD3 | Allergic airway inflammation/ asthma ^13^; lung fibroblast EMT ^14^; cell cycle arrest, apoptosis ^15^; lung fibrosis ^14^ |
| hsa-1228-3p_st | 7.23 | 7.83 | 1.51 | *0.009* | MOAP1 | Stress-induced apoptosis ^16^ |
| hsa-191-3p_st | 4.02 | 3.10 | -1.90 | *0.002* | CDK6, SATB1, MDM4, TIMP3 | Cell proliferation, migration ^17^; senescence ^17^; |
| miRNA, present in CS-EMP and absent in AC-EMP (p<0.01) | | | | | |  |
| hsa-miR-2052_st | 0.97 | 1.49 | 1.43 | *0.004* |  | Unreported |
| hsa-miR-4534_st | 1.02 | 1.42 | 1.31 | *0.005* | GSG1L,GNG13,HLA-DMB | B cell malignancy ^18^ |
| hsa-miR-196a_st | 0.85 | 1.23 | 1.30 | *0.006* | HOXB8, ARF4, MEF2A, CDK2 | Cancer prognosis ^19^ |
| hsa-miR-4510_st | 0.99 | 1.27 | 1.22 | *0.008* | KLK4, PAK3 , ZNF583 | B cell malignancy ^18^ |
| miRNA, most abundant, non-regulated by CS in EMP P>0.5) | | | | | |  |
| hsa-miR-3665_st | 11.38 | 11.43 | 1.03 | *0.54* | BOK, FUT6 , NDFIP1 | Apoptosis ^20^, cell proliferation and inflammation ^21,22^ |
| hsa-miR-3960_st | 11.16 | 11.19 | 1.02 | *0.67* | EN1, CERS1 | Osteoblast differentiation ^23^, Ceramide regulation |
| hsa-miR-4497_st | 10.90 | 11.12 | 1.17 | *0.54* | KCNA3 , SHOX, LRRC7, BRD8 | B cell malignancy ^18^ |
| hsa-miR-4787 5p_st | 10.50 | 10.44 | -1.04 | *0.63* |  | Unreported |

**Abbreviations:** ARF4: ADP-ribosylation factor 4; BOK, bcl2-related ovarian killer; BRD8, bromodomain-containing protein 8; CDC25A, cell division cycle 25A; CDK2, cyclin-dependent kinase 2; CDK6, cyclin-dependent kinase 6; CERS1, ceramide synthase 1; EMT, epithelial-mesenchymal transition; EN1, engrailed homeobox 1; FUT6, fucosyltransferase 6; GNG13, guanine nucleotide-binding protein; GSG1L, germ cell-specific gene 1-like protein; HLA-DMB, human leukocyte antigen beta chain; HMGA2, high-mobility group AT-hook 2; HOXB8, homeobox protein B8; IL-13, interleukin 13; KCNA3, potassium voltage-gated channel, shaker-related subfamily, member3; KLK4, kallikrein-related peptidase; LRRC7, leucine-rich repeat-containing protein 7; MEF2A, Myocyte enhancer factor 2A; MOAP1, modulator of apoptosis 1; NDFIP1, Nedd4 family-interacting protein 1; PAK3, p21 protein-activated kinase 3; SATB1, stabling-1; SHOX, short stature homeobox; SMAD3, mother against decapentaplegic homolog 3; TIMP3, metallopeptidase 3; ZNF583, zinc finger protein 583; All miRNAs are verified through miRBase^24^ and downstream targets searched through miRTarBase^25^ or TarBase^26^.

**Supplementary Table 3.** **miRNAs detected in mouse plasma cMPs and modulation by CS**

| Probeset ID | Mean (AC) | Mean (CS) | Fold (CS/AC) | *p-value (CS/AC);*  *FDR 0.34* | Major confirmed (or highly predicted) targets | Putative function |
| --- | --- | --- | --- | --- | --- | --- |
| miRNAs, most altered by CS exposure *(p<0.03)* | | | | | |  |
| mmu-143_st | 1.96 | 4.19 | 4.69 | *0.007* | MAPK7, MYO6, HK2, KRAS, FNDC3B, KLF4, DNMT3A, BCL2 | Smooth muscle cell fate, differentiation and plasticity; apoptosis; angiogenesis; ^27^ |
| mmu-126-3p_st | 4.24 | 6.15 | 3.74 | *0.025* | SPRED1, PI3KR2, VEGFA, HOXA9 | Endothelial-specific miR; angiogenesis, endothelial activation ^28,29^; inter-cellular regulation of VSM turnover ^30,31^; EPC proliferation, differentiation ^32^ |
| mmu-706_st | 5.17 | 6.75 | 3.00 | *0.022* | SLC3A2 | Unreported |
| mmu-2137_st | 8.24 | 5.88 | -5.16 | *0.013* | SPI1, PAX1 | Unreported |
| mmu-709_st | 5.90 | 3.38 | -5.74 | *0.028* | CTCFL, MYC, AKT1 | Oncogenesis |
| mml-miR-638_st | 8.06 | 7.45 | -1.53 | *0.021* | OSCP1 | Smooth muscle cell proliferation and migration ^33^ |
| miRNA, present in CS-cMP and absent in AC-cMP (p<0.04) | | | | | |  |
| mmu-miR-3110_st | 0.95 | 1.48 | 1.44 | *0.027* | PHC1, ADAM12, PDP2, HIP2, RAB37 | Unreported |
| mmu-miR-378-star_st | 1.05 | 1.44 | 1.32 | *0.007* | ODC1, SUFU | Mitochondrial metabolism ^34^; liver regeneration ^35^ |
| miRNA, most abundant, non-regulated by CS in cMP (p>0.1) | | | | | |  |
| mmu-miR-5128_st | 9.34 | 9.01 | -1.26 | *0.49* | ZMYND11, TCF4, FUT4, BNC2, RAPH1 | B-cell function ^36^ |
| mmu-miR-5126_st | 9.22 | 8.83 | -1.31 | *0.38* | LAG3, NUP50 | B-cell function ^36^ |
| mmu-miR-3960_st | 8.07 | 7.65 | -1.34 | *0.14* | POU3F3, ATM, PIM-1, SERT | Osteoblast differentiation ^23^ |
| mmu-miR-16_st | 8.06 | 8.36 | 1.24 | *0.46* | CCND17, VEGFA, WNT3A, MDM4 | Down-regulated in hypoxia, VEGF regulation ^37^ |

**Abbreviations:** ADAM12: Disintegrin and metalloproteinase domain-containing protein; ATM: Ataxia Telangiectasia Mutated; BCL2: B-Cell Lymphoma 2;BNC2: Basonuclin 2; CCDC117: Coiled-Coil Domain Containing 117; CTCFL: CCCTC-binding factor-like protein; DNMT3A: DNA (Cytosine-5-)-Methyl Transferase 3A; EPC: Endothelial progenitor cell; FNDC3B: Fibronectin type III domain containing 3B; FUT4: Fucosyltransferase 4; HIP2: Huntingtin-Interacting Protein-2; HK2: Mitochondrial Hexokinase II; HOXA9: Homeobox Protein A9; KLF4: Kruppel-like factor 4; LAG3: Lymphocyte-activation gene 3; MAPK7: Mitogen Activated Protein Kinase 7; MDM4: Mouse Double Minute 4; MYC: Myelocytomatosis Oncogene; MYO6: Myosin VI; NUP50: Nucleoporin 50; ODC1: Ornithine decarboxylase 1; OSCP1: Organic solute carrier protein 1; PAX1: Paired box 1; PDP2: Pyruvate dehydrogenase phosphatase catalytic subunit 2; PHC1: Polyhomeotic Homolog 1; PI3KR2: Phosphatidylinositol 3-Kinase Regulatory Subunit Beta; POU3F3: POU Class 3 Homeobox 3; RAPH1: Ras Association (Ralgds/AF-6) And Pleckstrin Homology Domains 1; SERT: Serotonin Transporter; SLC3A2: Solute carrier family 3 member 2; SOCS3: Suppressor Of Cytokine Signaling 3; SPI1: Spleen focus forming virus proviral integration oncogene; SPRED1: Sprouty-Related, EVH1 Domain-Containing Protein 1; SUFU: Suppressor of fused homolog; TCF4: Transcription Factor 4; VEGFA: Vascular endothelial growth factor A; VSM: Vascular smooth muscle; ZMYND11: Zinc Finger, MYND-Type Containing 11; All miRNAs are verified through miRBase ^24,38-41^ and downstream targets searched through miRTarBase ^25^ or TarBase ^26^.

**Supplementary Table 4.** **Ceramide content of EMP released in response to CS.**

| **Ceramides (fmol/μl)** | **CTL** | | **CS** | | **T-test**  (p value) |
| --- | --- | --- | --- | --- | --- |
|  | Mean | SD | Mean | SD |  |
| 14:0 | 0 | 0 | 6 | 8 | 0.2 |
| 16:0 | 23 | 15 | 210 | 41 | <0.001 |
| 18:1 | 0 | 0 | 1 | 2 | 0.2 |
| 18:0 | 0 | 0 | 50 | 73 | 0.2 |
| 20:0 | 0 | 0 | 5 | 5 | 0.1 |
| 24:1 | 3 | 6 | 37 | 4 | <0.01 |
| 24:0 | 3 | 6 | 47 | 41 | 0.2 |
| **Total ceramides** | 33 | 23 | 363 | 119 | <0.01 |
| **Total dihydroceramides** | 0 | 0 | 7 | 10 | 0.2 |

Ceramide species, total ceramides and total dihydroceramides content released from MLEC following CS exposure (10%; 24h; mean, SD, n=3,CTL; n=5,CS; Student’s t-test).

**Supplementary figures and legends**



Supplementary Figure 1. A. Abundance of circulating MPs (cMPs) in plasma of CS-exposed mice. Mean+SEM; ANOVA, p<0.01 (Dunnett’s *p<0.05 vs. AC, n=4-9). B. Abundance of EMPs released from MLEC exposed to AC- or CS extract (10%; 24h). Mean+SEM; t-test (*p<0.05, n= 3). **Inset**: Flow cytometry sizing of EMPs generated by CS-exposed MLEC against standard fluorescent polystyrene beads (outlined EMPs size <1 μm). **C-E.** Flow cytometry detection of exosome and apoptosis markers (Mean+SEM) on EMPs released from untreated or AC- or CS-exposed MLEC (10%; 24h). **C.** Histone (ANOVA p<0.05; Tukey’s *p<0.05, n= 3). **D**. Phosphatidyl serine detected by Annexin V staining (ANOVA p=0.001, Tukey’s *p<0.05, n= 3). **E**. CD63 (ANOVA p=0.01, Tukey’s *p<0.05, n= 3); **i-ii**. Relative abundance of CD63+ and Annexin V+ EMPs released from AC- or CS-exposed MLEC.

**Supplementary Figure 2. A-C.** Relative abundance (%) of circulating ceramide species in cMPs isolated from COPD plasma (**A**, mean of n=8); in total human plasma obtained from apparently healthy non-smokers (**B**, mean of n=3); or in total human plasma from subjects with COPD (**C**, mean of n=14). Note similar pattern of ceramide species abundance in cMPs and unfractionated plasma. Statistically significant differences (Student’s t test, p<0.05) are indicated for either increased (*) or decreased (#) ceramide species relative abundance in plasma of COPD compared to healthy subjects. **D-E.** Relative abundance (%) of intracellular ceramide species in HLMVEC cultured for 16h in either no serum- (**D**; mean; n= 3) or in regular serum-containing media (**E,** mean; n=4). Note the distinct pattern of ceramide species in the intracellular compartment of EMP compared to that of cMPs (in A) and its modification by the presence of FBS in the culture medium.

**Supplementary video 1.** Time lapse video (1h) of HLMVEC with plasma membrane visualized by red fluorescence (following transfection of RFP-plasma membrane CellLight construct) exposed to AC extract (5%). Note (at six o’clock) protruding lamellipodia.

**Supplementary video 2.** Time lapse video (1h) of HLMVEC with plasma membrane visualized by red fluorescence (following transfection of RFP-plasma membrane CellLight construct) exposed to CS extract (5%). Note initial retraction of cell body (at one and three o’clock) leaving filopodia footprints that during retraction release round submicron structures, presumably EMPs.

**Supplementary video 3.** Time lapse video (30 min) of HLMVEC with plasma membrane visualized by red fluorescence (following transfection of RFP-plasma membrane CellLight construct) exposed to CS extract (2%). Note (at 12 o’clock’) initial retraction of cell body leaving filopodia footprints that, during retraction, release round submicron structures, presumably EMPs.
